# Response of Alfalfa Leaf Traits and Rhizosphere Fungal Communities to Compost Application in Saline–Sodic Soil

**DOI:** 10.3390/microorganisms12112287

**Published:** 2024-11-11

**Authors:** Tian-Jiao Wei, Guang Li, Yan-Ru Cui, Jiao Xie, Zheng-Wei Liang, Fa-Chun Guan, Zhong-He Li

**Affiliations:** 1 Jilin Academy of Agricultural Sciences, China Agricultural Science and Technology Northeast Innovation Center, Changchun 130033, China; tianjiao25@126.com (T.-J.W.); lg-1207@163.com (G.L.); nkycyr@126.com (Y.-R.C.); 15584435748@163.com (J.X.); 2 Northeast Institute of Geography and Agroecology, Chinese Academy of Sciences, Changchun 130102, China; liangzw@iga.ac.cn

**Keywords:** compost, saline–sodic soil, rhizosphere fungal community, *Sodiomyces*, leaf photosynthetic traits

## Abstract

Soil salinization is considered a major global environmental problem due to its adverse effects on agricultural sustainability and production. Compost is an environmentally friendly and sustainable measure used for reclaiming saline–sodic soil. However, the responses of the physiological characteristics of alfalfa and the structure and function of rhizosphere fungal communities after compost application in saline–sodic soil remain elusive. Here, a pot experiment was conducted to explore the effect of different compost application rates on soil properties, plant physiological traits, and rhizosphere fungal community characteristics. The results showed that compost significantly increased soil nutrients and corresponding soil enzyme activities, enhanced leaf photosynthesis traits, and ion homeostasis compared with the control treatment. We further found that the rhizosphere fungal communities were dominated by *Sodiomyces* at the genus level, and the relative abundance of pathogenic fungi, such as *Botryotrichum*, *Plectosphaerella*, *Pseudogymnoascus*, and *Fusarium,* declined after compost application. Moreover, the α-diversity indexes of the fungal community under compost application rates of 15% and 25% significantly decreased in comparison to the control treatment. The soil SOC, pH, TP, and TN were the main environmental factors affecting fungal community composition. The leaf photosynthetic traits and metal ion contents showed significantly positive correlations with *Sodiomyces* and *Aspergillus*. The fungal trophic mode was dominated by Pathotroph–Saprotroph–Symbiotroph and Saprotroph. Overall, our findings provide an important basis for the future application of microbial-based strategies to improve plant tolerance to saline-alkali stress.

## 1. Introduction

Soil salinization is one of the major environmental problems that threatens agricultural production and limit crop yield [1]. Saline-alkali stress, distinguished by elevated levels of Na^+^ and pH, exerts more intricate effects on plants compared to salt stress [2,3,4]. Composting, a biochemical procedure transforming degradable organic matter into stable humus, stands out as a notable sustainable waste management solution [5]. Applying compost boosts soil nutrients, promotes microbial growth, and is vital for crop production and soil restoration [6,7]. In contrast, biochar production is complex and requires high-temperature pyrolysis, which increases costs. Compost can mature at low temperatures (below 80 °C), resulting in lower energy consumption while effectively enhancing soil organic matter and microbial activity [8]. Additionally, compared with manure, compost undergoes fermentation, which effectively eliminates pathogenic microorganisms and reduces pollution risks while significantly minimizing odors. It provides balanced nutrients, improves soil permeability and moisture retention, and reduces heavy metal accumulation, thus supporting sustainable agricultural development [9]. Overall, compost outperforms both biochar and manure in terms of safety, odor control, nutrient balance, and soil enhancement. Current studies have shown that compost leaches excess salt and reduces sodium content more efficiently than mineral fertilizers [10]. At the same time, it can effectively reduce pH value and induce the enrichment of beneficial plant microbial communities, thus improving the saline-alkali tolerance [11]. Compost offers soil microbes more accessible forms of carbon and nitrogen than biochar. This availability enhances the nutritional status of the soil and increases microbial metabolic activity [12].

The rhizosphere refers to the part of the microdomain environment that is different from the soil in physical, chemical, and biological properties due to the influence of plant root activities. Rhizosphere fungal communities play a key role in promoting soil nutrient cycling, enhancing plant stress tolerance and disease resistance [13]. For instance, arbuscular mycorrhizal fungi (AMF) can greatly enhance plant nutrient absorption efficiency and stress resistance by establishing a symbiotic relationship with plant roots [14,15]. *Penicillium malochii* and *Zygosporium masonii* can antagonize plant pathogens and encourage plant growth by protecting plants from diseases [16,17]. Moreover, some saprophytic fungi *Sordariales* and *Pezizales* can release nutrients, such as nitrogen, phosphorus, and trace elements, when they decompose compost or organic matter, which can be taken up and used by plants to promote plant growth and development [18,19]. The lack of diversity of fungal species in saline-alkali soil poses a significant challenge. One effective strategy for restoring these lands is to supplement with halophilic fungi, microorganisms that thrive in extreme saline-alkali conditions. These fungi can absorb salt ions, produce organic acids, and release cellulose-degrading enzymes, which help to mitigate saline-alkali damage while enhancing soil physical properties and fertility. Additionally, some halophilic fungi exhibit strong resistance to various stresses and can degrade crop straw, making them valuable tools for identifying genes associated with abiotic stress resistance and cellulose decomposition. By introducing halophilic fungi, we can improve the quality of saline-alkali soil and promote sustainable agricultural development [20]. Moreover, *Sodiomyces* is known for its antibiotic production [21] and the synthesis of polysaccharides in alkaline environments, aiding in the formation of extracellular polymers that support plant development [22]. *Aspergillus aculeatus* has the capability to enhance phosphorus and potassium levels crucial for plant growth [23] and can also generate bioactive compounds that enhance plant photosynthesis [24,25]. While numerous studies have highlighted the impact of organic amendments on soil fungal community structure, the outcomes are often inconclusive. The diversity of fungal communities has been observed to either decrease [19,26] or increase [27,28] following the application of organic amendments. However, the research focus on the structure and function of plant rhizosphere fungal community after compost application in saline–sodic soil is still limited.

Leaves serve as the primary organs utilized by the above-ground parts of plants for resource acquisition [29]. They produce essential nutrients for plant growth and development and facilitate resource exchange between plants and the external environment [30]. The varying characteristics of plant leaf morphology and biochemical traits can indicate the adaptability of plants to environmental fluctuations [31]. Rhizosphere growth-promoting bacteria and mycorrhizal fungi play a key role in enhancing plant adaptability to stressful environments, such as by increasing leaf area, chlorophyll content, and enhancing leaf nutrient absorption [32,33]. However, previous studies only focused on the relationship between plant roots and rhizosphere microorganisms but ignored the relationship between leaf traits and rhizosphere microorganisms [34,35]. Soil enzymes are recognized as sensitive indicators that mirror the metabolic activities of soil microorganisms, primarily originating from plant root exudates, microbial secretions, and organic litter degradation products. They play a vital role in the decomposition of organic matter and soil nutrient cycling [36]. Effective soil management practices have the potential to boost soil biological activity, mitigate microbial resource constraints [37,38], and influence soil microbial community structure, ultimately enhancing soil fertility and positively impacting crop productivity [39,40]. Therefore, exploring the response of soil microbial communities to soil nutrient cycling and plant growth and development is critical to understanding the mechanisms of agricultural soil organic remediation.

Alfalfa (*Medicago sativa* L.) is a perennial leguminous forage grass known for its high yield, quality, and robust salt tolerance [41]. Our previous research revealed that saline-alkali stress can diminish photosynthesis, disrupt ion balance, and significantly impede plant growth and development [3,4]. Nevertheless, the impact of compost application on the soil and plant rhizosphere microbiome in saline–sodic soil, particularly the correlation between the rhizosphere fungal community, soil environmental factors, and plant traits, remains uncertain.

In this study, we conducted a pot experiment to explore the response of growth and physiological characteristics and rhizosphere fungal communities of alfalfa seedlings to different compost application rates. Here, we aimed to (1) investigate the compost application on the growth characteristics of alfalfa, the properties of saline–sodic soil, and the diversity and structure of rhizosphere fungal communities; (2) elucidate the interaction among rhizosphere fungal communities, soil properties, and plant traits after compost application; and (3) predict the potential functions of rhizosphere fungal communities.

## 2. Materials and Methods

### 2.1. Pot Experiment Setup and Sample Collection

Alfalfa (*Medicago sativa* L. ‘Gongnong NO. 1’) seeds were planted in plastic pots (15.6 cm diameter × 15.5 cm depth). In our study, we utilized compost addition rates of 0%, 5%, 15%, and 25% that were adjusted based on the rates proposed by Liu et al. [42] and Mazumder et al. [40]. These treatments were identified as SA, SAF5, SAF15, and SAF25, respectively. The control soil (SA) was saline-alkali soil with 0% compost added and no other fertilizers added. The saline–sodic soil was collected (0–20 cm) from the Da’an Sodic Land Experimental Station (45°36′ N, 123°53′ E, and 132.1 m) in the Songnen Plain, Northeast China. The compost product used in this study was provided by Zhongsheng Environmental Protection Technology Development Co., Ltd. (Changchun, Jilin, China), and the compost products were fermented from corn stalks and chicken manure. The chemical properties of the soil and compost are shown in Supplementary Materials Table S1, and the fungal community composition in compost is shown in Supplementary Materials Figure S1.

All of the experiments were conducted with three biological replicates, each treatment consisting of 3 pots of alfalfa seedlings, with 15 seedlings in each pot. A controlled growth chamber was set to 25 °C day/20 °C night with a 12-h photoperiod at 350 μmol photons m^−2^ s^−1^ light intensity. During the growth period, all pots were adjusted to 60% field water capacity with tap water. The pots were rotated within the growth chamber every 1–2 days to minimize any effect of location.

After 8 weeks of growing alfalfa seedlings, the five seedlings under each treatment were taken as a group and the leaves collected from the plants were stored in an envelope. The rhizosphere soil was collected according to the method of Chang et al. [15]. Briefly, the roots were placed in a tube containing 5 mL of sterile water. A layer of soil (1 mm) surrounding the roots was collected by centrifuging for 30 s at a relative centrifugal force of 10,000× *g*. After removing the excess water, the soil from the rhizosphere was preserved on dry ice and then transferred to −80 °C. Furthermore, the loose soil surrounding the roots was separated into two portions: one part was air-dried and sifted through a 2 mm sieve to analyze the soil’s chemical properties, while the other part was stored at −4 °C to assess enzyme activity.

### 2.2. Measurement of Plant Traits and Soil Properties

The photosynthetic traits leaf soil and plant analyzer development value (SPAD), leaf nitrogen content (LN), and leaf water content (LW) were detected by the spectrum of chlorophyll analyzer (TYS-4N, Jinkelida Instrument Co., Ltd., Beijing, China). Specifically, the fully developed and expanded leaves of the third leaf from the top to the bottom were randomly selected from the plant to measure within 8:00–10:00 a.m. Each biological repetition consisted of five leaves. The blade temperature was set to 25 °C, the humidity to 55%, and the flow rate to 350 μmol s^−1^. Leaf area (LA) was scanned by ImageJ Version 1.53t software (http://imagej.nih.gov/ij accessed on 15 October 2024) [43]. Leaf fresh weight (LF) was measured using a balance (YP20002B, Lichenkeji Co., Ltd., Changsha, China). Then, the leaves were placed in an oven (DHG101S, Subo Corporation, Shaoxing, China), defoliated at 105 °C for 15 min, and then dried to a constant weight at 75 °C; finally, leaf dry weight (LD) was obtained using a balance (YP20002B, Lichenkeji Co., Ltd., Changsha, China). The leaf Na^+^ content and K^+^ content were determined as previously described using an inductively coupled plasma mass spectrometer (ICPS-7500, Shimadzu Corporation, Kyoto, Japan) [4]. The leaf K^+^/Na^+^ ratio was calculated.

Soil pH was measured using the potential method (water-to-soil ratio of 5:1) with a pH meter (DDS-307A, Shanghai Lei-Ci, Shanghai, China). The total nitrogen content (TN), total phosphorus content (TP), and soil organic carbon content (SOC) were analyzed following the procedure outlined by Wang et al. [2]. TN and TP levels were measured using a continuous flow analyzer (San++, SKALAR Corporation production, Breda, The Netherlands) after digesting soil samples with H_2_SO_4_-HClO_4_. SOC was quantified through dichromate oxidation with heating (K_2_Cr_2_O_7_–H_2_SO_4_) and subsequent back titration with 0.5 mol L^−1^ FeSO_4_. Soil β-glucosidase activity (BGC), urease activity (URE), and alkaline phosphatase activity (APC) were assessed using soil kits (UPLC-C-B935/UPLC-C-B933/UPLC-C-B900, Shanghai UPLC-MS Testing Technology Co., Ltd., Shanghai, China) as per the manufacturer’s guidelines. BGC, URE, and APC activities were determined through nitrophenol colorimetry, indophenol blue colorimetry, and phenylene disodium phosphate colorimetry, respectively. These activity indices were measured using a spectrophotometer (T6, Puxi General Instrument Co., Ltd., Beijing, China).

### 2.3. Sequence Processing and Bioinformatics Analysis

Soil DNA was isolated from a 0.25 g soil sample using the FastDNA^®^ SPIN Kit For soil Kit (MP Biomedicals, Solon, CA, USA). The fungal intergenic spacer ITS1 region was amplified using primer sets ITS1-F (GGAAGTAAAAGTCGTAACAAGG) and ITS1-R (GCTGCGTTCTTCATCGATGC). The sequencing was performed on the Illumina NovaSeq platform and 250 bp paired-end reads were generated. The datasets presented in this study can be found in the National Centre for Biotechnology Information (NCBI) Sequence Read Archive (SRA) under the accession number PRJNA1153319.

Microbiome bioinformatics was conducted with QIIME2 2019.4 [44], slightly modified based on the official tutorials (https://docs.qiime2.org/2019.4/tutorials/, accessed on 28 September 2022). Briefly, raw sequence data were demultiplexed using the demux plugin, then primers were cut with cutadapt plugin [45]. Next, the sequences underwent a series of processing steps: quality-filtered, denoised, merged, and chimera removed using the DADA2 plugin [46]. Non-singleton amplicon sequence variants (ASVs) underwent multiple sequence alignment through mafft [47] and species classification information was obtained through constructing the fasttree2 database [48]. The taxonomy assigned to the ASVs was annotated using a pre-trained Naive Bayes classifier from QIIME 2’s classify-sklearn algorithm, which references the UNITE database (Release 8.0) [49]. ASV-level alpha diversity indices, such as Pielou_e index, Shannon index, and Simpson index, were calculated with the diversity plugin. The Bray–Curtis and UniFrac distance metrics were visualized using principal coordinate analysis (PCoA), non-metric multidimensional scaling (NMDS), and unweighted pair-group method with arithmetic means (UPGMA) hierarchical clustering. These analyses were performed using the vegan package in R software (R Foundation for Statistical Computing, Vienna, Austria). Additionally, redundancy analyses (RDAs) of environmental variables and microbial community structure were performed using the same package. The volcano plot shows the average abundance of amplicon sequence variations (ASVs) in the fungal communities for different compost application rates compared to the control (SA). This difference analysis was performed using the DESeq2 package in R software [50]. ASVs were considered significantly different if they had a false discovery rate (FDR) below 0.05 and an absolute log_2_ fold change of 1 or greater. Additionally, a Venn diagram was used to illustrate the ASVs that were shared, as well as those particularly enriched or depleted across the different compost treatments. The shared and particularly enriched ASVs in different compost treatments were plotted in a Venn diagram. The linkages between fungal communities and leaf traits were evaluated using the Mantel test. The functional classes of these ASVs were predicted using FUNGuild [51].

### 2.4. Statistical Analysis

The test data were organized using Microsoft Excel 2016, and data plotting was conducted using Origin 9.1 software. Results were presented as the mean ± standard error. Statistical analyses were performed using SPSS Version 20.0 for Windows (SPSS, Chicago, IL, USA), utilizing one-way ANOVA followed by Duncan’s multiple range tests. Statistically significant results were defined as those with *p*-values < 0.05.

## 3. Results

### 3.1. Soil Properties and Plant Traits

Significant variations in soil nutrients were observed among different compost application rates, with SOC ranging from 2.94 to 29.30 g kg^−1^, TN ranging from 0.37 to 4.29 g kg^−1^, and TP ranging from 0.41 to 1.74 g kg^−1^ (Table 1). Under each compost addition ratio treatment, soil pH was significantly decreased compared to the control group, while soil BGC, soil URE, and soil APC were significantly higher than those in the control group (*p* < 0.05); and between different compost addition ratio treatments, soil BGC and soil URE showed a decreasing trend with an increase in compost addition rate. However, soil APC decreased first and then increased. Compared with the control treatment, LF and LD under each compost addition ratio were significantly increased (*p* < 0.05), and the fresh weight and dry weight of leaves under SAF15 treatment were the largest, which were 3.91 times and 4.07 times of SA in the control group, respectively.

Compared to the control group, the levels of LA (leaf area), SPAD (leaf soil and plant analyzer development value), LN (leaf nitrogen content), and LW (leaf water content) in alfalfa seedlings showed significant increases with varying rates of compost addition (*p* < 0.05). Notably, SPAD, LN, and LW were the highest in the seedlings treated with SAF15 (Table 2). The SA increased by 43%, 33%, and 35%, respectively, compared to the control treatment. With the increase in compost addition rate, leaf Na^+^ content (LNa^+^) showed a decreasing trend and reached the lowest (3.95 mg g^−1^) under SAF25 treatment, while the leaf K^+^ content (LK^+^) showed an increasing trend, and reached the highest (49.28 mg g^−1^) under SAF25 treatment (Table 2).

LA, SPAD, LN, and LW showed a significantly negative correlation with LNa^+^; meanwhile, SOC, TN, TP, URE, and APC showed a negative correlation with LNa^+^. LW, LK^+^, and LK^+^/Na^+^ showed a significantly positive correlation with LA (Figure S1).

### 3.2. Illumina Run Metrics

After filtering, a total of 1,352,427 ITS sequences were retained, with an average length of 243 bases. The ASV abundance table was processed to 95% of the minimum sample sequence size as the flattening depth, allowing us to obtain the relative abundance of observed ASVs for each sample at this sequencing depth. In total, 2028 ASVs were obtained from the 12 soil samples, with each sample containing between 210 and 309 ASVs. A total of 835 ASVs were identified in the FUNGuild database for subsequent analysis.

### 3.3. Rhizosphere Fungal Community Composition and Diversity

**Figure 1:**
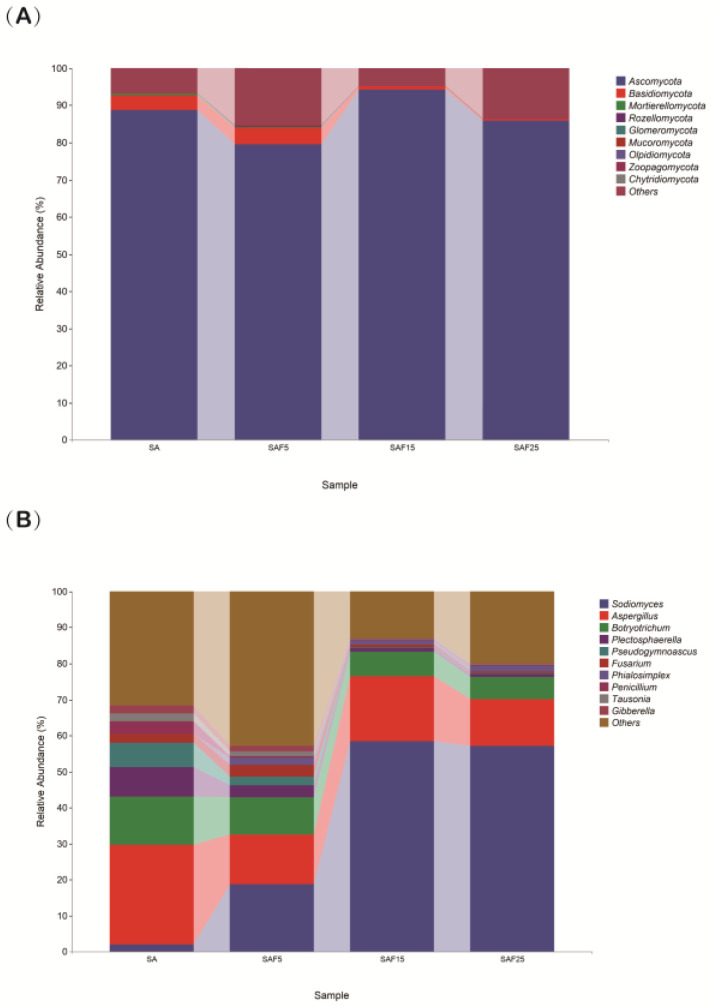
Effects of compost on rhizosphere fungal community at phylum level (**A**) and genus level (**B**) in alfalfa. The SA, SAF5, SAF15, and SAF25 represent different compost application rates, respectively.

Principal component analysis (PCA) showed that the rhizosphere fungal communities of alfalfa treated with SAF5, SAF15, and SAF25 were far apart from those treated with SA. Among them, the distance between SAF15 and SAF25 treatment is close, indicating that compost application will change the community of saline-alkali microorganisms. At the same time, the soil microbial composition under the low compost addition ratio was not similar to that under the medium and high compost addition ratio (Supplementary Materials Figure S2). The cumulative contribution of X axis (90%) and Y axis (8.3%) reached 98.3%, indicating that compost could significantly change the microbial composition of saline-alkali soil. The soil fungal communities were mainly composed of *Ascomycota*, *Basidiomycota*, and *Mortierellomycota* at the phylum level, accounting for approximately 90% of total sequences, whereas *Ascomycota* (58.4%) and *Basidiomycota* (21.9%) dominated the root fungal community (Figure 1A). SAF15 showed the highest relative abundance of the phylum *Ascomycota* (94.38%), and SAF25 showed the lowest relative abundance of the phylum *Basidiomycota* (0.32%) and *Mortierellomycota* (0.07%). The soil fungal communities were dominated by *Sodiomyces*, *Aspergillus,* and *Botryotrichum* at the genus level, which accounted for approximately 43% of all sequences. Notably, *Sodiomyces* exhibited the highest relative abundance (58.44%) in SAF15 treatment, while *Aspergillus* and *Botryotrichum* showed the lowest relative abundance of 12.89% and 6.20% in SAF25 treatment (Figure 1B).

**Figure 2:**
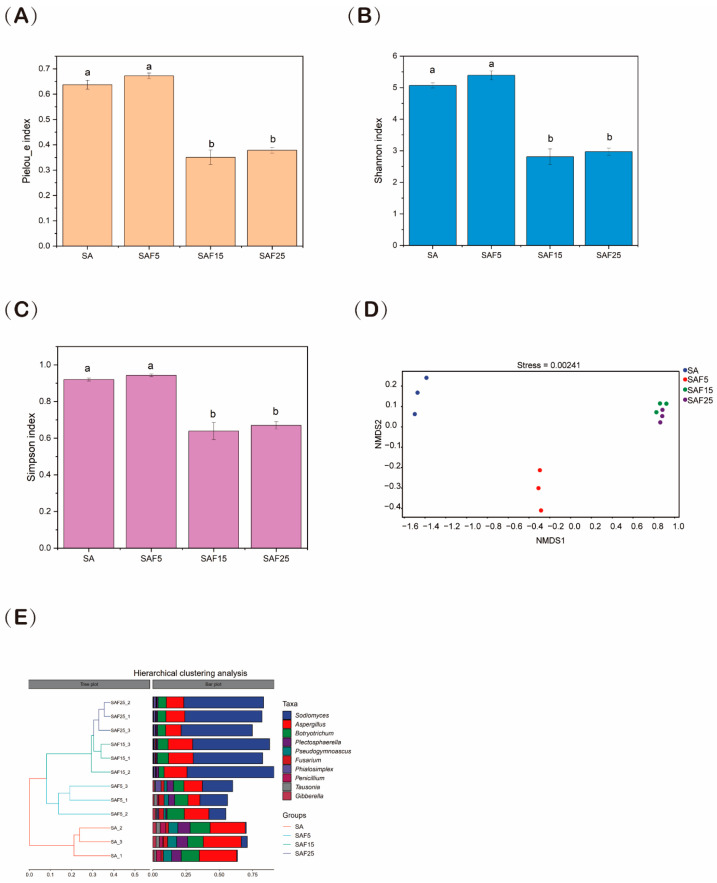
Effects of compost on alpha diversity Pielou_e index (**A**), Shannon index (**B**), and Simpson index (**C**). NMDS (Non-metric Multidimensional Scaling) plot (**D**) based on Bray−Curtis dissimilarity of fungal communities. The hierarchical tree (**E**) shows the UPGMA in soil under different treatments. The SA, SAF5, SAF15, and SAF25 represent different compost application rates, respectively.

Compared with CK, the alpha diversity indices (Shannon, Pielou_e, and Simpson) of the fungal communities in SAF15 and SAF25 treatments significantly decreased (*p* < 0.05) (Figure 2A–C). NMDS analysis of fungal beta diversity indicated that the microbial community was mainly separated between SA and SAF5 treatments at the first NMDS axis, whereas the effect of SAF15 and SAF25 was embodied in the second NMDS axis (Figure 2D). Hierarchically clustered genus-level heatmap analysis was used to identify high-dimensional biomarker taxa with significantly different abundances under different compost application treatments (Figure 2E). As the proportion of compost application rates increased, *Sodiomyces* gradually became the dominant genus, followed by *Aspergillus*. *Botryotrichum*, *Plectosphaerella*, *Pseudogymnoascus*, and *Fusarium* were less abundant in SAF15 and SAF25 compared with SA.

### 3.4. Linkages Between Rhizosphere Fungal Community and Soil Environmental Factors

**Figure 3:**
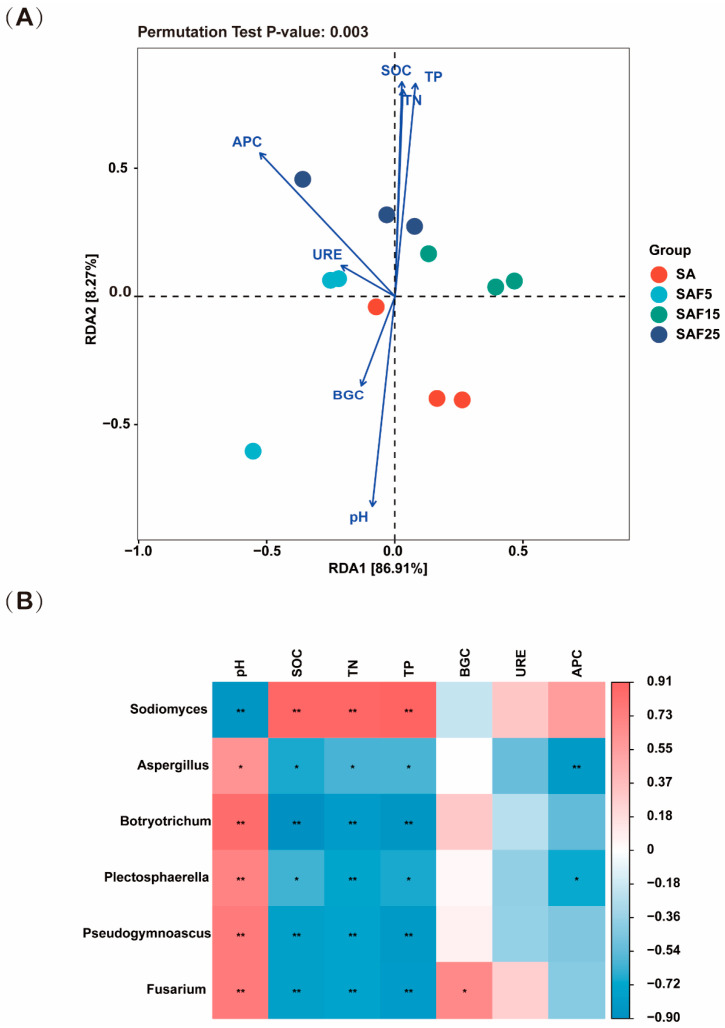
Redundancy examination of the soil properties and the fungal microbial communities at phylum level (**A**). Heatmap of Spearmans’ rank correlation coefficients of the top 6 fungal genus-level relative abundance and soil properties (**B**). Note: The horizontal row represents soil chemical properties, the vertical row represents microbial community abundance information, blue represents negative correlation, red represents positive correlation, the darker color indicates a higher correlation, and the *p*−value is the correlation test result (* *p* < 0.05, ** *p*< 0.01). SOC, soil organic carbon content; TN, total nitrogen content; TP, total phosphorus content; BGC, β-glucosidase activity; URE, urease activity; APC, acid phosphatase activity.

Redundancy analysis (RDA) was used to identify the environmental factors that lead to changes in fungal community structure (Figure 3A). The eigenvalues of the first and second axes were 86.91% and 8.27%, respectively. The cumulative percentage of variance data of species showed that the first two RDA axes explained 98.23% of the structural changes. The soil pH, TP, TN, and SOC were the main environmental factors affecting the differentiation of fungal communities.

The correlation results showed that environmental factors were more strongly associated with the relative abundance of fungi at the genus level. Specifically, SOC, TN, and TP showed significantly positive correlations with *Sodiomyces* (*p* < 0.01), but the above three indices showed significantly negative correlations with *Botryotrichum* (*p* < 0.01), *Pseudogymnoascus* (*p* < 0.01), and *Fusarium* (*p* < 0.01), *Aspergillus* (*p* < 0.05), *Plectosphaerella* (*p* < 0.05), and *Fusarium* (*p* < 0.01). The pH showed an opposite pattern of correlation with these fungi genera. APC showed significantly negative correlations with *Aspergillus* (*p* < 0.01) and *Plectosphaerella* (*p* < 0.05) (Figure 3B).

### 3.5. Relationships Between Rhizosphere Fungal Communities and Plant Traits

**Figure 4:**
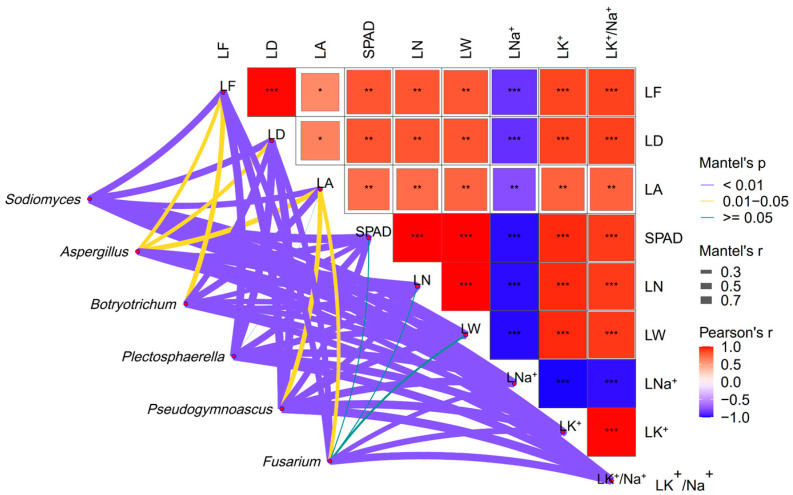
Mantel test analysis of correlations between rhizosphere fungi composition (at the genus level) and leaf traits. *, *p* < 0.05; **, *p* < 0.01; ***, *p* < 0.001.

Mantel test further analyzed the correlation between microbial composition and plant traits. For example, LNa^+^, LK^+^, LK^+^/Na^+^, LN, LW, and SPAD showed significantly positive correlations with *Sodiomyces* (*p* < 0.01) and *Aspergillus* (*p* < 0.01), and *Plectosphaerella* (*p* < 0.01). LF and LD showed significantly positive correlations with *Sodiomyces* (*p* < 0.01), *Aspergillus* (*p* < 0.05), *Botryotrichum* (*p* < 0.01), *Plectosphaerella* (*p* < 0.01), *Pseudogymnoascus* (*p* < 0.01), and *Fusarium* (*p* < 0.01) (Figure 4).

### 3.6. Prediction Potential Functions of Rhizosphere Fungal Communities

**Figure 5:**
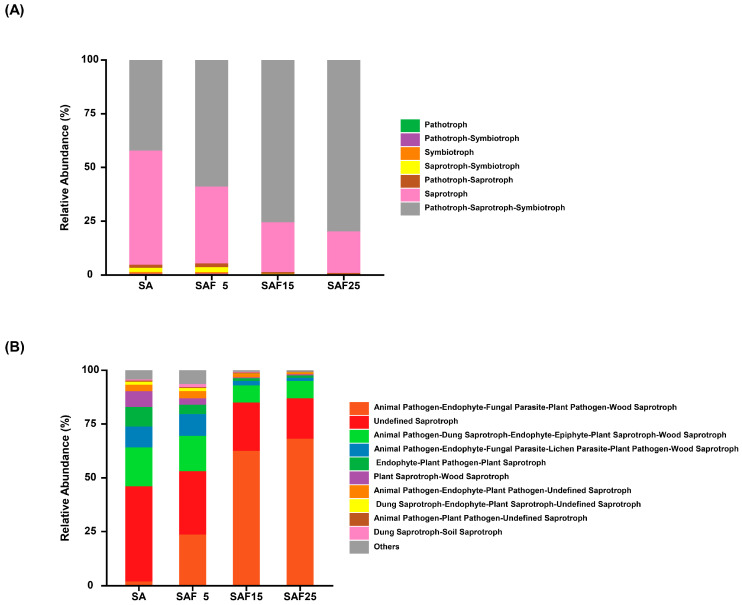
FUNGuild annotated fungal trophic modes (**A**) and main fungal functional guilds (**B**) in different compost application rates. The SA, SAF5, SAF15, and SAF25 represent different compost application rates, respectively.

The FUNGuild tool was used to predict the rhizosphere fungal community of different treatment groups, and seven fungal trophic modes were identified. The fungal trophic modes were dominated by Pathotroph–Saprotroph–Symbiotroph and Saprotroph (Figure 5A, Supplementary Materials Table S2). The fungal abundance of Pathotroph–Saprotroph–Symbiotroph showed an increasing trend with the increase in compost addition rate. The maximum value was 79.78% under SAF25 treatment. Nevertheless, the abundance of Saprotroph presented an opposite result. On the other hand, the most abundant ecological group in the rhizosphere soil of alfalfa was Animal Pathogen–Endophyte–Fungal Parasite–Plant Pathogen–Wood Saprotroph, with abundance ranging from 23.66% to 68.21%, followed by Undefined Saprotroph, with abundance ranging from 18.67% to 29.43% (Figure 5B).

**Figure 6:**
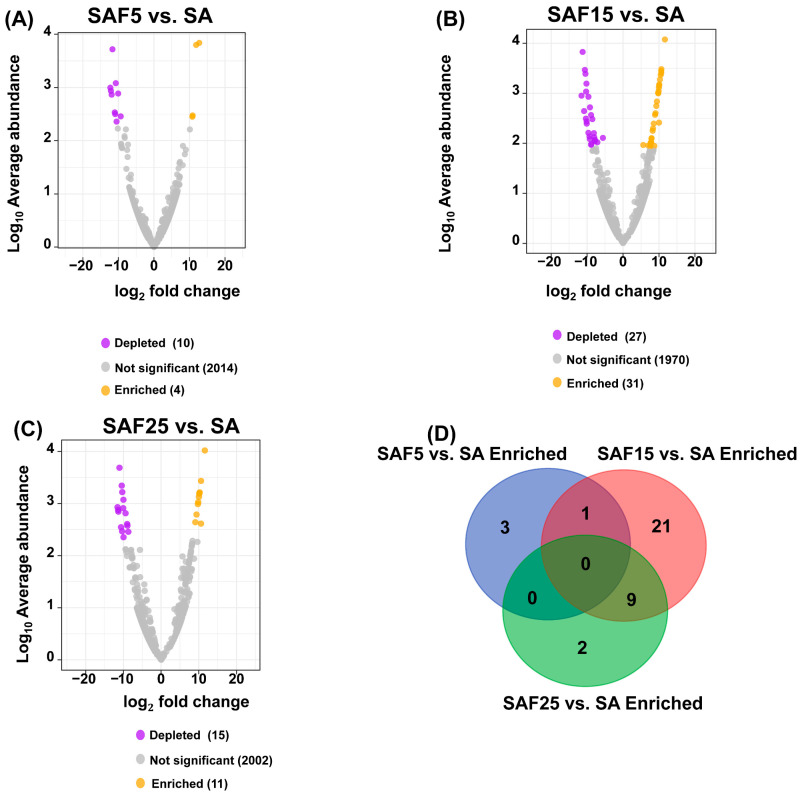
Volcano plot of the average abundance of non-singleton amplicon sequence variants (ASVs) for different compost application rates compared to SA (**A**–**C**). Common and specific enriched ASVs (**D**) under different comparison groups were plotted in the Venn diagram. The SAF5 vs. SA, SAF15 vs. SA, and SAF25 vs. SA represent different comparison groups, respectively.

We further performed the difference analysis of fungal communities based on different compost addition treatments relative to control treatment to determine the ASVs strongly affected by different compost rates (Figure 6A–C). Three comparison groups were created: SAF5 vs. SA, SAF15 vs. SA, and SAF25 vs. SA. In the three comparison groups, 14 (4 Enriched and 10 Deleted), 58 (31 Enriched and 27 Deleted), and 26 (11 Enriched and 15 Deleted) differentially expressed genes (DEGs) were identified, respectively. The number of enriched ASVs in SAF15 treatment was the highest, followed by those in SAF25 treatment and SAF5 treatment (Figure 6D). Interestingly, the significantly enriched ASVs (ASV_1165, ASV_389, ASV_129, ASV_1678) belonging to genus *Sodiomyces* were involved in the Pathotroph–Saprotroph–Symbiotroph function. The significantly enriched ASVs (ASV_1281, ASV_1837, ASV_699) belonging to genus *Aspergillus* were involved in the Saprotroph function (Supplementary Materials Table S3).

## 4. Discussion

### 4.1. Compost Enhanced Plant Adaptability to Sodic-Alkaline Stress

Saline–sodic soil usually has a high salt content and high pH, which can limit water and nutrient uptake by plants, thus affecting plant growth [3]. Plants exposed to environmental stresses endure morphological and physiological alterations [31]. In this study, leaf growth and physiological traits, such as LF, LD, SPAD, LA, LN, and LW, in saline–sodic soil were significantly higher than those in the control group under different compost application rates (Table 2). The presence of colloidal particles in organic matter within compost helps to absorb water, reducing evaporation losses [52]. At the same time, compost boosts important enzyme activities related to soil carbon (C), nitrogen (N), and phosphorus (P), which facilitates nutrient cycling for plant uptake [53]. Increased nitrogen levels promote plant growth and improve photosynthetic efficiency, resulting in higher leaf SPAD values. Additionally, applying compost encourages the exchange of sodium (Na) ions for potassium (K) ions, which reduces soil salinity and helps alleviate oxidative stress in plants [54]. This process leads to an increased accumulation of osmoprotective agents in plants, enhancing their tolerance to salt and alkali conditions [10]. Additionally, the presence of compost treatment enhanced the microbial viable biomass and its activity by supplying easily accessible carbon substrates that initiate the microbial metabolism crucial for carbon cycling [12]. Compost may raise the amount of organic nitrogen (N) in soils, which may improve net primary production by acting as a slow-release fertilizer [55]. In general, compost application can improve the salinized soil environment, provide more soil nutrients, promote soil microbial activity, regulate soil pH value, increase the resilience of plants, and thus, enhance the growth adaptability of plants to saline-alkali stressed environments [56].

### 4.2. Compost Induced Alfalfa to Recruit Specific Rhizosphere Fungal Communities

Fungi are crucial microbial components in soil ecosystems, playing key roles in essential ecological functions, including decomposition, parasitism, pathogenesis, and symbiosis [57]. The type and amount of fertilizer (e.g., inorganic and organic) can directly affect the function and structure of soil fungal communities [19,26]. In this study, Ascomycota was predominant at the phylum level in the rhizosphere after compost application (Figure 1A). This result is supported by other studies, *Ascomycota* is the most abundant phylum of fungi and is the main saprophytic trophic fungi in soil. Moreover, saprophytic fungi are active primary or secondary decomposers; they promote the mineralization process and change the nutrient supply rate and resource allocation in soil [58,59,60]. An increase in the abundance of saprophytic fungi may lead to increased niche sharing between plant species and a greater use of limiting nutrients, thus promoting plant growth [61]. It has been reported that the species found in almost every soda soil sample is *Sodiomyces*, an obligate alkalophilic ascomycete [62]. Intriguingly, our study showed that *Sodiomyces* was dominant at the genus level with increasing compost addition rates (Figure 1B), consistent with the previous findings, suggesting that fertilizer application regulates changes in soil microbial diversity and encourages the development of *Sodiomyces*. This could be attributed to the ability of *Sodiomyces* to produce membrane lipids and cytoplasmic sugar content [22,63], generate trehalose and arabitol [64], or synthesize antimicrobial compounds [21] to adapt to alkaline environments. Organic amendments directly stimulate cellular defense or initiation, causing greater defense induction after triggering pathogen infestation [65]. Numerous plant pathogens, such as *Fusarium* [66] and *Plectosphaerella* [67], inflict substantial harm on a wide array of crops through their pathogenicity and virulence factors. As expected, our results found that the abundance of these potentially dangerous fungi decreased compared with control, which may also lower the risk of plant diseases (Figure 1B). Mehta’s research has validated that the disease resistance mechanism of compost primarily encompasses the following factors: microbial population competition, antibiosis, hyperparasitism, systemic acquired resistance (SAR), induced systemic resistance (ISR), prevention of pathogen proliferation, and the physicochemical properties of compost [68]. In forthcoming studies, it is suggested to isolate the beneficial fungal communities from alfalfa in saline soil conditions and delve deeper into the mechanisms of disease inhibition.

Furthermore, in the present study, the α-diversity indexes of the fungal community in rhizosphere soil of alfalfa seedlings with more than 15% compost addition, such as Shannon index, Pielou_e index, and Simpson index, significantly decreased (Figure 2A–C). This was consistent with the studies of Jin et al. and Du et al., wherein the addition of exogenous organic fertilizer decreased the α-diversity index of soil fungi [19,69]. This may have been due to the fact that adding compost introduces large amounts of organic matter and nutrients into the soil, which might have led to increased resource competition among rhizosphere fungi. Some fungi may use these resources more efficiently, leading to a decrease in the number and diversity of others [70]. Moreover, increasing the ratio of compost in the soil may suppress microbial activity. Research indicates that excessive fertilizer usage can lead to a decrease in fungal diversity and biomass [71], thereby weakening the contribution of fungal communities to the soil ecosystem functions [72]. Although other studies have reported that organic amendments can increase soil fungal communities, the conclusions reached are largely dependent on the differences in test design, soil type, test years, and selected indicators used in the experiment [27,28]. This study is based on the results of laboratory simulation experiments, and the applicability of the experiment needs to be further verified in field trials in the future.

The FUNGuild functional prediction reveals that the Pathotroph–Saprotroph–Symbiotroph and Saprotroph nutritional mode predominates in the SA and different compost treatment rates. Recent studies reported that fertilization also significantly altered fungal composition by altering major nutrient patterns [73,74]. Organic fertilizers strongly affect microbial communities by transferring oligotrophic organisms adapted to nutrient-limited soils to specific microbial communities that decompose complex organic compounds [18]. In the current study, the relative abundance of Animal Pathogen–Endophyte–Fungal Parasite–Plant Pathogen–Wood Saprotroph increased relative to SA and proportionally to the amount of compost. Organic amendments can alleviate organic carbon hunger, thereby regulating microbial community interactions and stability as they provide an expanded food base [65]. In addition, the Undefined Saprotroph (undefined saprotroph) function group of fungi was not classified, indicating that the fungal community is complex and still needs to be further explored.

### 4.3. Compost Mediated Rhizosphere Fungal Community–Soil–Plant Interactions

The soil nutrient status is widely recognized as a critical determinant influencing the composition of soil fungal communities [75,76]. Research indicates that the use of external fertilizers can alter the environmental ecological niche of soil and drive the evolution of soil fungal communities [13,77]. In line with these findings, our study demonstrates that soil organic carbon (SOC), pH, total phosphorus (TP), and total nitrogen (TN) are key environmental factors shaping the composition and structure of fungal communities post-compost application (Figure 3A). Numerous studies suggest that soil pH plays a significant role in microbial community distribution by affecting soil nutrient availability [78]. In this study, after adding 25% compost, the soil pH decreased from 9.12 to 8.04, and the α-diversity of rhizosphere microbial community decreased significantly. Howell et al. reported that the pH of the rhizosphere microbial community decreased from 8.47 to 7.86 by adding 5% compost in saline-alkali soil, and the change trend of α-diversity was the same as this study. It can be seen that the slight change in soil pH caused by adding compost in saline-alkali soil has an impact on the α-diversity index [79]. Soil carbon content serves as a nutrient and energy source for fungal growth, playing a vital role in fungal reproduction and metabolic processes [80]. Moreover, soil fungi exhibit high sensitivity to soil nitrogen and phosphorus levels [81]. Increased nitrogen and phosphorus content in soil can stimulate arbuscular fungi colonization, modify soil pH, and impact leaf carbon, nitrogen, and phosphorus ratios [82]. In summary, our findings indicate that the addition of compost reduces soil pH, enhances major carbon, nitrogen, and phosphorus nutrients, and creates a more conducive environment for fungal survival. Monitoring and managing these factors can facilitate the targeted regulation of soil fungal communities.

The relationship between rhizosphere fungal community function and plant growth is multifaceted, encompassing activities such as nutrient cycling, pathogen inhibition, and soil structure conditioning, all of which collectively promote plant growth and health [14,15]. Our findings revealed significant positive correlations between leaf photosynthesis traits and *Sodiomyces* (Figure 4). This could be attributed to the ability of *Sodiomyces* to produce polysaccharides in alkaline environments, aiding in the formation of extracellular polymers that support plant development [22,63], or to generate cytosol-soluble carbohydrates like trehalose and arabitol, adapted to alkaline conditions [64]. *Sodiomyces* can produce antimicrobial compounds that protect plants from diseases [21]. In addition, according to the results of the difference analysis, we found that the rhizosphere fungus *Sodiomyces* was significantly enriched after adding compost (Figure 6). Interestingly, *Sodiomyces* exists in the original saline-alkali soil (SA treatment), but it is not the dominant genus of fungi in compost (Supplementary Materials Figure S1), and the relative abundance of *Sodiomyces* shows an upward trend under the application rates of 5–15% compost. In contrast, the relative abundance of *Sodiomyces* at 25% was lower than that at 15% (Figure 1B), which indicated that the regulation of compost on recruiting *Sodiomyces* from alfalfa roots to cope with saline-alkali stress was a delicate interaction process of plants, soil, and microorganisms.

It is worth noting that we observed how important parameters involved in leaf photosynthesis traits, such as SPAD, LN, and LW, were significantly positively correlated with *Aspergillus*. In alignment with the study by Li et al. [23], *Aspergillus* has been shown to improve nutrient uptake and forage quality in bermudagrass by enhancing the availability of phosphorus and potassium. Potassium and phosphorus levels are known to impact photosynthetic rates [83]. As a P-solubilizing microorganism, *Aspergillus* can convert insoluble phosphorus into a bioavailable form through various solubilization and mineralization mechanisms [84]. Similarly, *Aspergillus* is recognized as a K-solubilizing microorganism that plays a role in converting insoluble potassium forms into directly absorbable and utilizable forms for plants [85]. Moreover, *Aspergillus* can produce bioactive compounds such as auxin and gibberellin, which contribute to plant growth and photosynthesis benefits [24,25]. It is noteworthy that leaf traits exhibited notably positive correlations with significant plant pathogens like *Botryotrichum*, *Plectosphaerella*, *Pseudogymnoascus*, and *Fusarium*. Akanmu’s findings supported the notion that plants might elevate metabolic activity following pathogen invasion to bolster the immune system and facilitate the repair of damaged tissues following organic amendments [86]. Further investigation in future experiments is warranted to validate the connection between plant traits and the rhizosphere fungal community.

The structure and diversity of the soil microbial community are susceptible to environmental factors (temperature, salt, or water stress) and human factors (type, dosage, and application period of organic amendments) [87,88]. Studies conducted by Steven Heisey et al. reveal that a single compost application can have enduring effects on soil nitrogen levels, plant growth, and the composition of soil microbial communities, even under low-input conditions [89]. Similarly, research by Sadet-Bourgeteau et al. indicates that prolonged organic waste product (OWP) application can sustainably boost soil microbial biomass and impact microbial community structure [90]. Compost application directly enhances soil nutrients and fungal communities, with repeated use offering lasting benefits. Continuous organic material input fosters nutrient accumulation, promoting fungal growth and diversity. Over time, fungal communities may evolve through competitive interactions, leading to the dominance of certain species through ecological adaptation. This dynamic process contributes to enhanced soil health and productivity. Therefore, exploring the enduring effects of repeated compost application is crucial for a comprehensive understanding of its advantages.

## 5. Conclusions

In summary, our findings revealed that compost can enhance the growth adaptability of alfalfa in saline–sodic soil by inducing specific rhizosphere fungal communities to be recruited. We found that compost enhanced soil enzyme activities, including BGC, URE, and APC, and increased soil nutrients, including SOC, TN, and TP. Compost also increased the relative abundance of the predominant fungi *Sodiomyces*, and decreased the relative abundance of pathogenic fungi such as *Botryotrichum*, *Plectosphaerella*, *Pseudogymnoascus*, and *Fusarium*. However, the α-diversity indexes of the fungal community were significantly decreased after the compost application rate by 15% and 25%. The soil SOC, pH, TP, and TN were the main environmental factors affecting the fungal communities. The leaf photosynthetic traits and leaf metal ion showed significantly positive correlations with *Sodiomyces* and *Aspergillus*. Overall, the findings point to the potential effectiveness of compost application in restoring saline–sodic soil. Future studies, however, have to concentrate on evaluating these revisions’ viability and suggested adoption rates. The size of the treated area and the most effective techniques for adding amendments to the soil should be considered during this evaluation. Testing with several sample sites throughout time might also help to determine the long-term consequences of implementing these adjustments.

## Figures and Tables

**Table 1:** Response of soil chemical properties and soil enzyme activities to compost in saline–sodic soil.

Treatment	pH	SOC (g kg^−1^)	TN (g kg^−1^)	TP (g kg^−1^)	BGC (U g^−1^)	URE (U g^−1^)	APC (nmol^−1^ d^−1^ g^−1^)
SA	9.12 ± 0.04 a	2.94 ± 0.07 d	0.37 ± 0.01 d	0.41 ± 0.00 d	29.55 ± 0.55 c	305.76 ± 0.82 d	5047.15 ± 31.40 d
SAF5	8.93 ± 0.01 b	8.84 ± 0.07 c	0.86 ± 0.02 c	0.66 ± 0.02 c	39.45 ± 1.12 a	587.65 ± 4.28 a	7519.55 ± 28.22 b
SAF15	8.48 ± 0.01 c	18.58 ± 0.14 b	2.13 ± 0.09 b	1.18 ± 0.05 b	34.87 ± 0.58 b	548.15 ± 6.34 b	6512.30 ± 52.04 c
SAF25	8.04 ± 0.01 d	29.30 ± 0.29 a	4.29 ± 0.17 a	1.74 ± 0.08 a	24.84 ± 0.67 d	416.05 ± 4.99 c	7803.25 ± 47.26 a

SOC, soil organic carbon content; TN, total nitrogen content; TP, total phosphorus content; BGC, β-glucosidase activity; URE, urease activity; APC, acid phosphatase activity. All values are shown as mean ± standard error. Different lowercase letters indicate significant differences among compost treatments at *p* < 0.05 by Duncan’s multiple range test.

**Table 2:** Response of alfalfa leaf traits to compost in saline–sodic soil.

Treatments	LF (g^−1^)	LD (g^−1^)	LA (cm^2^)	SPAD	LN (mg g^−1^)	LW (%)	LNa^+^ (mg g^−1^)	LK^+^ (mg g^−1^)	LK^+^/Na^+^
SA	0.16 ± 0.01 d	0.03 ± 0.00 d	1.00 ± 0.07 b	28.71 ± 0.29 b	11.75 ± 0.09 b	46.57 ± 0.19 b	9.23 ± 0.20 a	23.28 ± 0.22 d	2.52 ± 0.03 d
SAF5	0.32 ± 0.04 c	0.06 ± 0.01 c	1.28 ± 0.18 ab	39.47 ± 0.62 a	15.15 ± 0.20 a	60.19 ± 1.17 a	5.39 ± 0.04 b	38.72 ± 0.35 c	7.18 ± 0.02 c
SAF15	0.62 ± 0.03 a	0.11 ± 0.00 a	1.48 ± 0.09 a	41.17 ± 0.89 a	15.67 ± 0.28 a	62.69 ± 1.11 a	4.12 ± 0.06 c	46.61 ± 0.30 b	11.31 ± 0.17 b
SAF25	0.47 ± 0.02 b	0.09 ± 0.00 b	1.50 ± 0.10 a	40.91 ± 0.65 a	15.60 ± 0.21 a	62.40 ± 0.82 a	3.95 ± 0.44 c	49.28 ± 0.31 a	12.49 ± 0.07 a

LF, leaf fresh weight; LD, leaf dry weight; LA, leaf area; SPAD, soil and plant analyzer development value; LN, leaf nitrogen content; LW, leaf water content; LNa^+^, leaf Na^+^ content; LNa^+^, leaf K^+^ content; LK^+^/Na^+^, leaf K^+^/Na^+^ ratio. All values are shown as mean ± standard error. Different lowercase letters indicate significant differences among compost treatments at *p* < 0.05 by Duncan’s multiple range test.

## Data Availability

The datasets for this study can be found in online repositories. The names of the repository/repositories and accession number(s) can be found at NCBI—PRJNA1153319.

## Supplementary Materials

The following supporting information can be downloaded at: https://www.mdpi.com/article/10.3390/microorganisms12112287/s1, Table S1. The basic chemical properties of soil and compost; Table S2. Prediction of potential functions of rhizosphere fungal communities; Table S3. Prediction potential functions of enriched non-singleton amplicon sequence variants (ASVs) under different comparison groups; Figure S1. Relative abundance of fungi at the level of genus in compost; Figure S2. Pearson correlation heatmap of soil properties and leaf traits; Figure S3. Principal component analysis (PCA) of rhizosphere fungal community of alfalfa.
