# Supplementary material for: Response of Alfalfa Leaf Traits and Rhizosphere Fungal Communities to Compost Application in Saline–Sodic Soil
Source: Microorganisms. 2024 Nov 11;12(11):2287. doi: 10.3390/microorganisms12112287 (PMC11596975; doi:10.3390/microorganisms12112287)
Supplement: Supplementary file 1 [file microorganisms-12-02287-s001.zip › Supplementary figures.pdf]

Supplementary Material

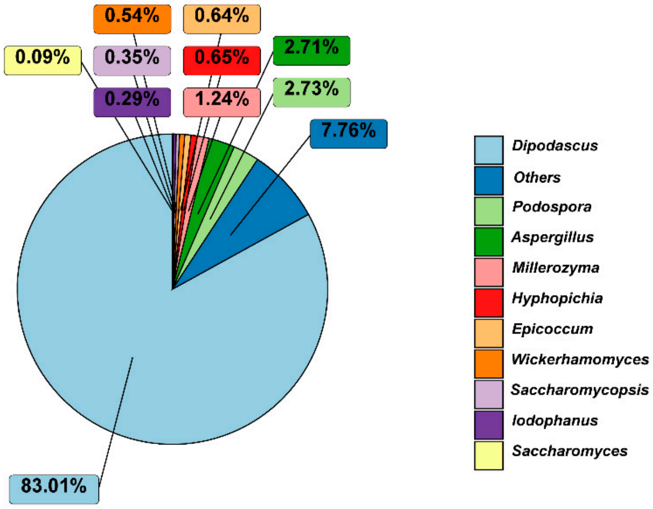

Figure S1. Relative abundance of fungi community at the level of genus in compost.

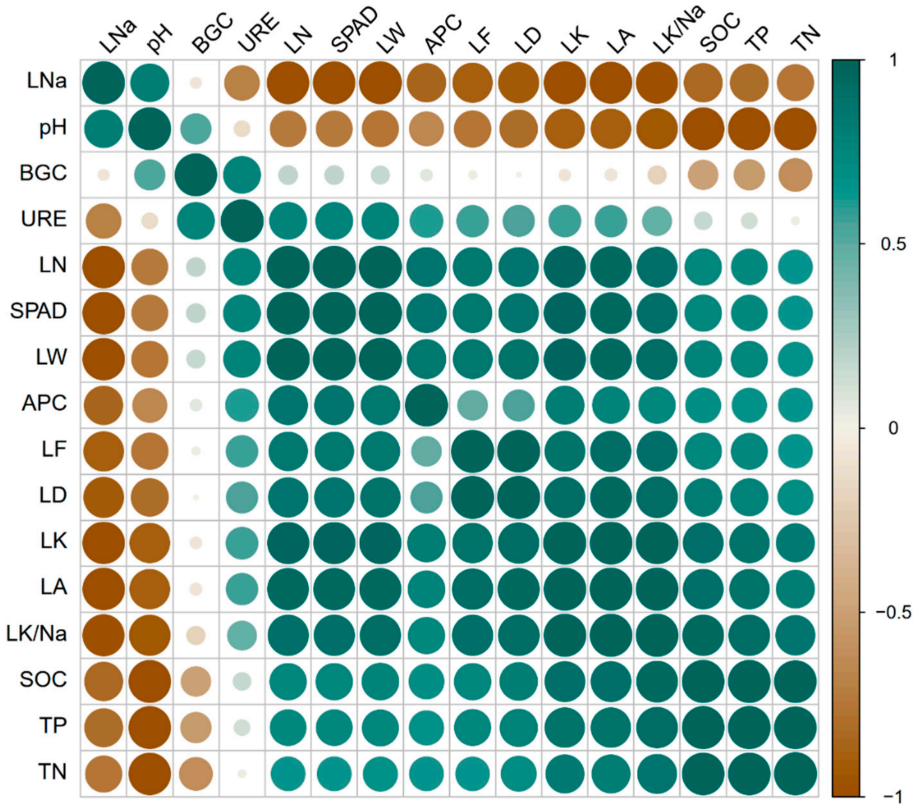

Figure S2. Pearson correlation heat map of soil properties and leaf traits.

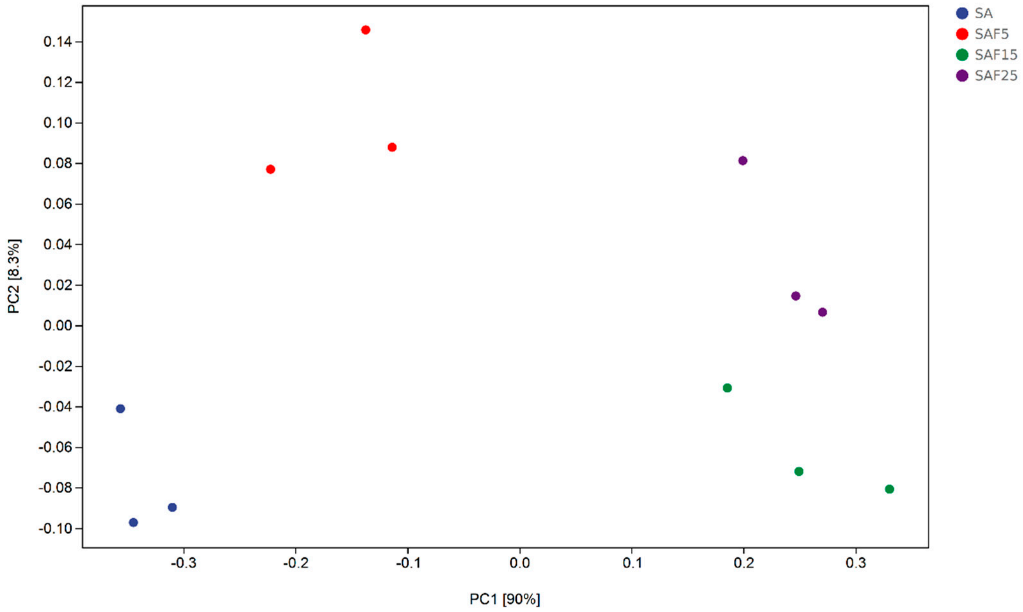

Figure S3. Principal component analysis (PCA) of rhizosphere fungal community of alfalfa.
